# Elastography of Endometrium in Women Taking Tamoxifen: A New Approach to an Old Diagnostic Problem

**DOI:** 10.3390/jcm9123870

**Published:** 2020-11-28

**Authors:** Krzysztof Jabłoński, Łukasz Kurek, Maciej Żukowski, Natalia Data-Jabłońska, Karolina Żukowska, Anna J. Milewska, Aleksandra Lipka, Marcin Jóźwik

**Affiliations:** 1 Clinic of Gynecology, Oncological Gynecology and Obstetrics, Municipal Polyclinical Hospital in Olsztyn, Niepodległości Str. 44, 10-045 Olsztyn, Poland; jabkrys@op.pl (K.J.); lukasz.kurek@uwm.edu.pl (Ł.K.); maciej.zukowski@uwm.edu.pl (M.Ż.); natared@o2.pl (N.D.-J.); 2 Department of Oncology and Immunooncology, Hospital Ministry of the Interior and Administration with Warmia and Mazury Oncology Center, Olsztyn, Wojska Polskiego Ave. 37, 10-228 Olsztyn, Poland; karolina.gornowicz@wp.pl; 3 Department of Statistics and Medical Informatics, Medical University of Białystok, Białystok, Szpitalna Str. 37, 15-295 Białystok, Poland; anna.milewska@umb.edu.pl; 4 Department of Gynecology and Obstetrics, School of Medicine, Collegium Medicum, University of Warmia and Mazury in Olsztyn, Żołnierska Str 18, 10-561 Olsztyn, Poland; aleksandra.lipka@uwm.edu.pl

**Keywords:** elastography, endometrium, endometrial cancer, endometrial cancer screening, SERM therapy, the softest endometrium layer, tamoxifen, ultrasonography

## Abstract

Tamoxifen is a commonly used selective estrogen receptor modulator applied in the treatment for breast cancer. However, in the endometrium, Tamoxifen stimulates tissue growth, cellular transformation, the migration of the cells, and metastatic potential in endometrial cancer. Considering that uterine cancer is the most common neoplasm of the reproductive tract and the third most common neoplastic disease in women, the aim of this study was to investigate if applying elastography in examining the endometrium was beneficial for uterine cancer screening protocols in women on selective estrogen receptor modulator therapy. This study was based on the execution of a classic assessment of the endometrium that included the evaluation of the following: echogenicity, central endometrial stripe, presence of fluid in the uterine lumen, myometrium–endometrium interface, intensity of vascularization and vascular pattern. An ultrasound presentation was then processed and analyzed with elastography. The values of the elastography parameters demonstrated good consistency for the measurement of the softest endometrial layer thickness in elastography. A strong positive correlation (R = 0.56) was demonstrated between the endometrial thickness, as determined by ultrasound examination, and the softest endometrial layer in elastography (*p* < 0.001). The research showed that the elastography measurements of the width of the softest endometrium layer, based on a population of women taking Tamoxifen, appeared to be a promising option for endometrial cancer screening.

## 1. Introduction

Breast cancer is the most common neoplastic disease in women and the most frequent cause of death among women affected by it in developed countries. In 2015, in Poland, 18,106 new breast cancer cases were reported. The current treatment protocol for breast cancer is multidisciplinary, and includes surgery, chemotherapy, radiation therapy, immunotherapy and hormonal treatment. Despite the evolving medical approach, the morbidity in breast cancer patients is increasing [1].

A reduction in the incidence of hormone-dependent neoplasms among the female population has been effected by treatment and prophylaxis with selective estrogen receptor modulators (SERMs). According to the Food and Drug Administration (FDA), the indications for SERM therapy for breast cancer include both adjuvant therapy and treatment for metastases. Tamoxifen is the most investigated and widely used selective estrogen receptor modulator applied in the treatment of breast cancer. In the mammary gland, SERMs demonstrate antiestrogenic activity, but their effects on the estrogen receptors found in the uterus are agonistic, i.e., they stimulate the endometrium to grow, which is a well-documented risk factor for endometrial cancer [2]. The impact of Tamoxifen on the endometrium most probably involves the activation of the PI3/AKT/mTOR pathway, which influences the estrogen receptor alpha (ERα), stimulating insulin-like growth factor 1 (IGF1) [2,3]. Tamoxifen also impacts the overexpression of the *AGR2* gene, which is associated with worse prognoses. These changes resulting from the agonistic effects of Tamoxifen on the estrogen receptors in the endometrium stimulate tissue growth, cellular transformation, the migration of the cells and the metastatic potential in endometrial cancer [4]. Tamoxifen also has an impact on a higher incidence of benign pathologies of the endometrium, such as endometrial polyps [5]. Considering that uterine cancer is the most common neoplasm of the reproductive tract and the third most common neoplastic disease in women after breast cancer and bronchial cancer, in some developed countries, the rationale behind the uterine cancer screening protocol in women on SERM therapy is being widely discussed [6,7,8,9,10].

Some studies indicate that the elevated incidence of endometrial cancer in women on the Tamoxifen treatment protocol is dependent upon the treatment time [11]. The risk of uterine neoplastic disease occurrence increases specifically in women who have been taking Tamoxifen for more than five years. It is also important to distinguish between the effects of the medication in premenopausal and postmenopausal women. Among premenopausal females, this drug does not demonstrate an enhanced estrogenic effect in the uterus, because its stimulating impact on endometrial growth is balanced by endogenous progesterone production. In contrast, it is estimated that the risk of Tamoxifen-induced endometrial cancer is between 2–7 times higher in postmenopausal patients [12]. Some reports have documented a higher incidence of sarcomas in Tamoxifen-treated patients, and a warning note released by the American College of Obstetricians and Gynecologists (ACOG) has been included in the usage notes accompanying the drug [8]. Despite the well-documented increase in the incidence of uterine neoplastic diseases, treatment with Tamoxifen has not been shown to lead to increased mortality for these reasons [8].

Transvaginal ultrasonography (US) is currently the basic diagnostic modality for the investigation of the endometrium, and such examination seems essential, particularly in postmenopausal women with symptomatic bleeding from the reproductive tract, as statistically, 10% of them are affected by cancer of the uterine body. Patients with a history of reproductive bleeding, in whom the endometrial thickness is 5 mm or more, should be diagnosed with an invasive approach: by uterine curettage and a microscopic examination of the collected sample. If the US thickness of the endometrium is less than 5 mm, it is not mandatory to undertake any invasive procedure. In premenopausal asymptomatic women, if the endometrium is 10 mm or less in thickness and no abnormalities are detected using endometrial ultrasonography, it is not compulsory to undertake invasive diagnostics [7,13]. This approach applies to all female patients who do not carry risk factors apart from taking Tamoxifen. Besides measuring the endometrial thickness, it is necessary to examine the echogenicity of the endometrium, the presence of fluid in the uterine cavity, the interface between the endometrium and the myometrium and, importantly, the nature of the vascular bed in the endometrium [6].

As US screening does not reduce mortality and there is a frequent risk of unjustified invasive diagnostics due to the typical US presentation of the endometrium during Tamoxifen therapy, the scientific consensus is to not recommend performing an obligatory US examination in women treated with Tamoxifen. The consensus of the ESMO-ESGO-ESTRO associations, released in 2015, recommends educating postmenopausal female patients treated with Tamoxifen about the higher risk of endometrial hyperplasia and uterine cancer [6]. Moreover, the consensus does not recommend performing a routine US screening procedure of the endometrium in asymptomatic or Tamoxifen-treated patients. The ACOG and SOGC also recommend educating Tamoxifen-treated women about the risk of endometrial hyperplasia, endometrial cancer and uterine sarcomas [7,8]. Bleeding from the reproductive tract should be considered an indicator for the need of further diagnostics. Postmenopausal women should undergo more detailed monitoring for symptoms of hyperplasia and cancer of the endometrium. Thus, a US examination of the endometrium does not fulfil the diagnostic needs of Tamoxifen-treated patients [6,7,8].

Elastography is a new radiological tool with an increasing scope of applications in medicine. An elastography exam involves an ultrasound performed according to the International Endometrial Tumor Analysis (IETA) guidelines. The US presentation is then processed with appropriate software to map the elastic properties and stiffness of the soft tissue. This method has been successfully applied in practical diagnostics for diseases of the thyroid gland, liver and mammary gland. Also, preliminary research revealed that elastography may be a valuable tool for differentiating endometrial pathologies from normal or atrophic endometria in women with endometrium thickness above 5 mm, as detected by a transvaginal ultrasound examination [14,15].

In 2013, the European Federation of Societies for Ultrasound in Medicine and Biology (EFSUMB) [16] and, in 2015, the World Federation for Ultrasound in Medicine and Biology (WFUMB) [17] released recommendations on the application of elastography, although they did not include elastography diagnostics of the endometrium, even though in the selected groups of patients that presented diagnostic challenges for 2D US, elastography could have been a valuable addition to standard diagnostic procedures.

Two techniques for elastography investigations have been implemented to date: dynamic study and static study. In brief, dynamic elastography determines the elasticity of the tissue by measuring the velocity with which a transverse wave percolates; this wave is produced acoustically, generated by an ultrasound device. Static elastography, in contrast, examines the distortion of the tissue when an external force (pressure) is applied. The latter technique is more widespread, and its physical mainstay is based on Hook’s law, stating that the Young’s modulus of linear deformability is proportionally dependent on stress and is in inverse proportion to elasticity. The changes in deformation of the tissues are processed using a color map with assigned numerical values, called the elastography-to-B-mode size ratio, and presented in the B model [16,18]. Mechanical differences between the endometrium and myometrium, and specifically, differences between the deformability of endometrial lesions and that of normal tissue, make this modality relevant in transvaginal ultrasound examinations in locations where access to the uterus is not limited by other organs, and when the urinary bladder is emptied. Thus, the application of elastography is warranted in examinations of the endometrium, in particular in Tamoxifen-treated patients for whom the standard transvaginal ultrasound US presentation of the endometrium overestimated its thickness. Therefore, the aim of this study was to compare the 2D US and elastography properties of the endometrium in Tamoxifen-treated patients to determine possible associations which may be of use for the development of an endometrial cancer screening tool.

## 2. Materials and Methods

### 2.1. Study Design

In order to recruit patients for the study, cooperation was established with the Oncology Clinic at the Hospital Ministry of the Interior and Administration with Warmia and Mazury Oncology Center in Olsztyn. The main inclusion criteria were peri- or post- menopausal period, and taking Tamoxifen for the chemoprevention of breast cancer. The exclusion criterion was taking Tamoxifen for less than six months. A letter of invitation, including a description of the study, was sent to 200 patients. The letter also included instructions on how to sign up for the examination. The reporting rate was set at 34.5%. After arriving at the Clinic of Gynecology, Oncological Gynecology and Obstetrics, Municipal Polyclinical Hospital in Olsztyn, patients confirmed their willingness to take part in the research by signing an informed consent. Each patient underwent a medical history examination, including information about coexisting diseases and risk factors for endometrial cancer. Then, after the patient emptied the urinary bladder, a 2D US and elastography with a transvaginal probe was performed. The data obtained during the examination was placed in the appropriate form, made in duplicate—one for the patient and the other for analysis. The trial was approved by the Bioethics Committee of the Faculty of Medicine, University of Warmia and Mazury in Olsztyn, on 22 June 2017 (Resolution No. 31/2017).

### 2.2. Description of the Investigated Patients

The study was carried out in the Clinic of Gynecology, Oncological Gynecology and Obstetrics, at the Municipal Polyclinical Hospital in Olsztyn, in 2018–2019. Sixty-nine female patients were treated for breast cancer with Tamoxifen, according to the adjuvant chemotherapy protocol, and were included in the trial (Table 1).

None of the subjects had pathological bleedings from the reproductive tract. Seven subjects were qualified for further invasive diagnostics with hysteroscopy and resulting fractional uterine curettage due to an ultrasound-measured endometrial thickness of over 11 mm; the collected material was investigated and verified with histopathology. 

### 2.3. Methodology of Elastography

**Figure 1:**
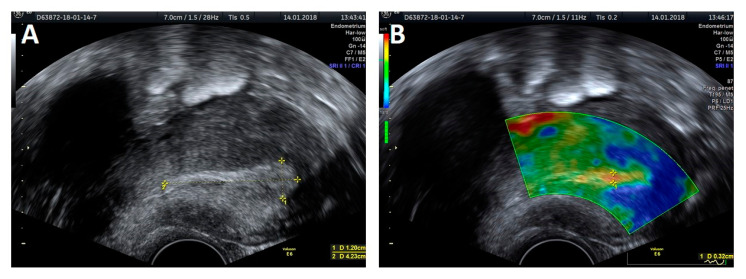
A 2D ultrasound presentation: endometrial thickness (**A**) and elastography: thickness measurement of the softest layer of the endometrium (**B**).

**Figure 2:**
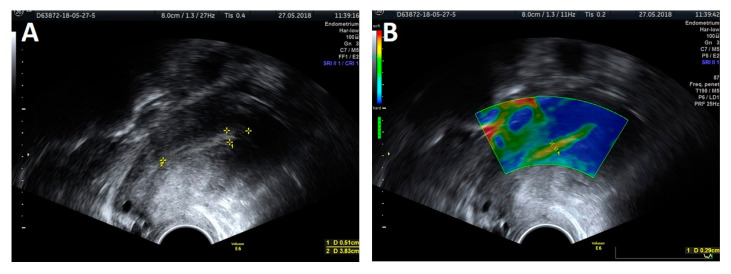
A 2D ultrasound presentation: endometrial thickness (**A**) and elastography: thickness measurement of the softest layer of the endometrium (**B**).

The trial was performed with a Voluson E6 GE (General Electric, Zipf, Austria) device, equipped with a transvaginal probe operating at 2.8–10 MHz, using the elastography strain modality. Apart from the thickness measurement, a classic evaluation of the endometrium was performed, based on criteria developed by the IETA group, that included endometrial and intracavitary lesions echogenicity, evaluation of the central endometrial stripe, the presence of fluid in the uterine lumen, evaluation of the myometrium–endometrium interface, the intensity of vascularization and the vascular pattern (Figure 1A and Figure 2A). An ultrasound presentation was then processed and analyzed with elastography. The softest area of the endometrium (the point with lowest elastography index), the myometrium–endometrium interface (the junction between the mucous membrane and smooth muscle layer) and endometrial contours (the outline of the softest endometrium area) were defined as the reference zones for the elastography indices (Figure 1B and Figure 2B). The reference point for setting the relative values of the elastography indices was the hardest area of the posterior myometrial wall, which was assigned a value of 1. Each area specifying the given elastography index was first assessed by the investigators on a color scale by assigning points, which were then summed to produce the total value for the elastography properties of the endometrium. The thickness of the softest endometrial area in the elastography presentation measured in the AP projection was also evaluated. The same examination was also carried out on 11 subjects by two independent investigators to validate the method.

### 2.4. Statistical Analysis

The normal distribution was verified with the Kolmogorov-Smirnov test with the Lilliefors’ modification and the Shapiro-Wilk test. A nonparametric Mann-Whitney U test was applied by comparing two quantitative variables without normality of distribution. A Wilcoxon signed-rank test was used for a comparison of two dependent variables without normality of distribution. The coefficient of Spearman’s rank correlation was calculated. Statistically significant results were recorded at *p* < 0.05. The statistical calculations were generated with the Statistica 13 software package (Statsoft, Tulsa, OK, USA) and the Stata/IC 12.1 software (StataCorp LP, College Station, TX, USA).

### 2.5. Validation of Measurements

The intraclass correlation coefficient (ICC; individual) for the two-way mixed-effects model and the 95% confidence interval for this coefficient were calculated to analyze the consistency of agreement for the measurements carried out by the first and second physician.

The following interpretation of ICC was developed:

- ICC < 0.5—poor consistency/reliability
- 0.5 ≤ ICC < 0.75—average consistency/reliability
- 0.75 ≤ ICC < 0.9—good consistency/reliability
- ICC ≥ 0.9—excellent consistency/reliability

## 3. Results

### 3.1. Elastography Measurements

The protocol of endometrial investigation with elastography and a transvaginal probe was applied in all 69 patients (Figure 1 and Figure 2). 

In seven females, hysteroscopy with subsequent fractional uterine curettage was performed as part of the qualification procedure for invasive diagnostics. In two cases, invasive diagnostics revealed the presence of endometrial polyps. In five cases, despite observing a wide endometrium (over 11 mm) in 2D ultrasound and a much lower value in the elastography, the hysteroscopy revealed an atrophy, which was confirmed by a histopathological examination.

**Figure 3:**
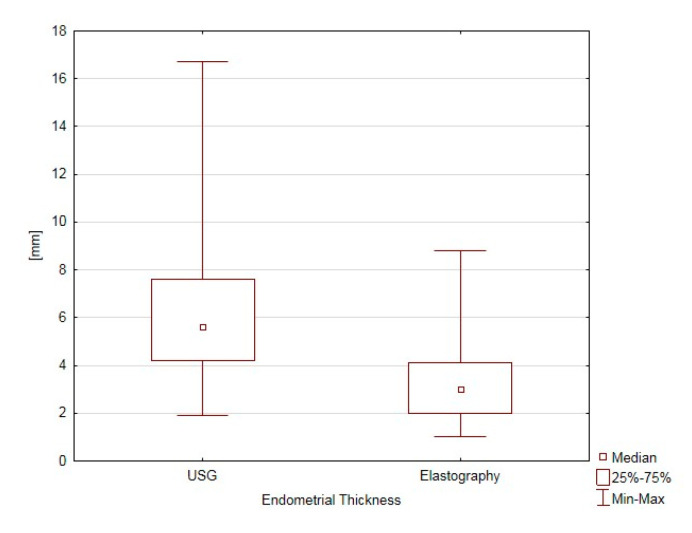
Comparison of endometrial thickness (ET) in ultrasound examination and thickness of the softest endometrial layer in elastography (TEE).

A statistically significant difference in the endometrial thickness was recorded between the ultrasound measurement with a transvaginal probe and the thickness of the softest endometrial layer in elastography (*p* < 0.001) (Figure 3).

**Figure 4:**
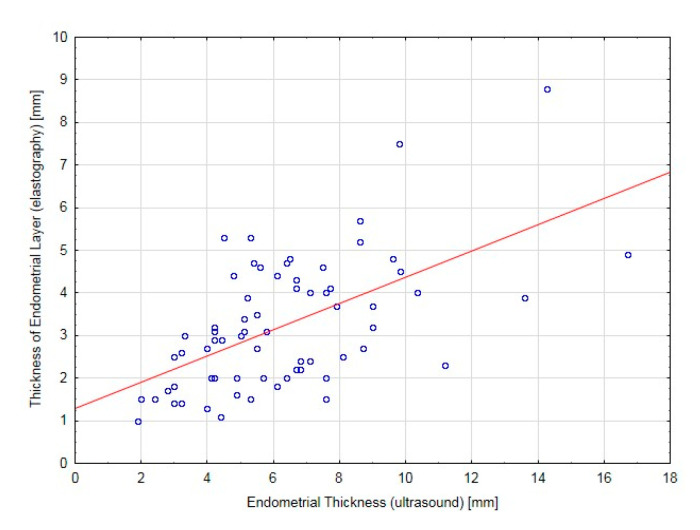
Scatterplot for endometrial thickness (ET) in the ultrasound examination and thickness of the softest endometrial layer in elastography (TEE).

The median value for the standard measurement of the endometrial thickness was 5.6 mm, whereas it was 3.0 mm for the softest endometrial layer in the elastography in the AP measurement (Figure 3). The median of the softest endometrial point index was 0.32 (reference range: 0.05–0.75), the index for the interface layer between the endometrium and myometrium was equal to 0.50 (with the reference range 0.16–0.86), and the contour of the softest endometrial layer index median was 0.40, with reference values from 0.12 to 0.95. When the trial was carried out by two investigators, attempts were made to validate the elastography modality for the measurement of the real endometrial thickness in the Tamoxifen-treated patient group. The values of the elastography parameters produced in the trial demonstrated good consistency only for the measurement of the thickness of the softest endometrial layer in elastography, for which ICC was 0.78. A strong positive correlation (R = 0.56) was demonstrated between the endometrial thickness in the ultrasound examination and the softest endometrial layer in elastography (*p* < 0.001) (Figure 4); this indicated that a correlation exists between these two parameters, In Tamoxifen-treated patients, the 2D US measurement of the width of the endometrium led to overestimations. As the parameters are positively associated, elastography makes it possible to accurately determine the width of the endometrium.

### 3.2. Evaluation of Validation

Statistically significant agreement of the measurements, carried out by investigators 1 and 2, was recorded for the ultrasound endometrial thickness, the thickness of the softest endometrial layer in elastography, the elastography index for the softest endometrial layer, the elastography index for the endometrium contour, the length of the uterine cavity, the AP dimension of the uterine cavity and the lateral dimension of the uterine cavity (Table 2).

The intraclass correlation coefficient showed excellent repeatability for the ultrasound endometrial thickness (ICC = 0.97), the length of the uterine cavity (ICC = 0.92) and the AP dimension of the uterine cavity (ICC = 0.97), and good repeatability for the thickness of the softest endometrial layer in elastography (ICC = 0.78), the elastography index for the softest endometrial layer (ICC = 0.75) and the lateral dimension of the uterine cavity (ICC = 0.77). In contrast, the intraclass correlation coefficient for the elastography index for the endometrium contour (ICC = 0.73) showed average repeatability, and poor repeatability for the elastography index for the endometrium–myometrium interface (ICC = 0.43).

Neither the ultrasound examination nor the elastography revealed any correlation between endometrial thickness and BMI, the duration of the treatment with tamoxifen or the length of the postmenopausal period.

## 4. Discussion

Tamoxifen is a proven survival-extending agent in patients treated for breast cancer. Recent studies have demonstrated that five-year therapy with Tamoxifen can be extended by another five years, giving a beneficial antagonistic effect by preventing relapse. Due to the agonistic effect of Tamoxifen on the endometrium and the increased risk of endometrial cancer, reliable investigations of the endometria of these patients are becoming an important element in health care [12,19]. In 2D ultrasound examinations, it can be difficult to differentiate between the actual thickness of the endometrium and the uterine stroma [13]. Since the standard ultrasound approach does not produce the desired outcome of a reliable diagnosis of endometrial hyperplasia or endometrial cancer, an ultrasound examination does not improve the effects of treatment with Tamoxifen for endometrial pathologies. This observation has already been made by scientific associations that do not recommend ultrasound screening in these patients [6,7,8], highlighting the need for new diagnostic options for these women. Recent gynecological studies postulated the diagnostic value of elastography in uterine disorders [14,15,20]. Among elastic scores, estimating the strain ratio gives the opportunity to distinguish among endometrial pathologies, as atrophic and normal endometrial tissues are the softest among all pathologies, followed by increasing strain ratio in endometrial polyps and hypertrophy [14]. Tumorous tissues are usually stiffer than healthy tissue; this observation was confirmed in endometrial cancer, in which malignant cervical lesions demonstrated higher strain ratios than benign pathologies [20,21]

Previous studies concerning elastography in a specific group of Tamoxifen-treated patients indicated that, in cases with endometrial pathologies, the endometrial tissue strain ratios were significantly increased (stiffer tissue, decreased elasticity) [22]. This study sought to develop a scoring system for differential diagnoses of endometrial changes [22]. We applied a different approach, as the numerical values of the elastography indices were dependent upon the examination conditions, technique and equipment characteristics. Furthermore, they were not fully repeatable for two different investigators. The repeatability was reported for the thickness of the softest endometrial layer in elastography in the uterine anteroposterior presentation. The color scale, which reflected the numerical values of the elastography indices, did not allow us to determine a repeatable range of colors, and thus, did not allow us to generate absolute numerical values that could provide an elastography description of the endometrium. To this end, the term “thickness of the softest endometrial layer in elastography” was used, which, despite the differences in the numeric indices, was repeatable in the individual investigations. The median of the thickness of the softest endometrial layer in elastography was statistically lower than that observed in the 2D ultrasound. Histopathological examinations did not reveal any pathologies in the patients qualified for invasive diagnostics due to the endometrial thickness in the 2D US. In these patients, the thickness of the softest endometrial layer in elastography in the anteroposterior presentation was significantly lower than that measured using standard ultrasound imaging; this information might have prevented qualifying these patients for invasive diagnostics. At the same time, this value was strongly correlated with the endometrial thickness measured with a 2D ultrasound transvaginal probe, which indicated the high reliability of endometrial investigation with elastography. Therefore, examination of the endometrium with elastography may become a new tool for the clinical assessment of women who are receiving prophylactic hormonal therapy for breast cancer with Tamoxifen. 

The results of the present trial show that the elastography measurement of the thickness of the softest endometrial layer may help to reduce the number of unnecessary invasive diagnostic examinations, such as hysteroscopy and fractional uterine curettage, in patients treated with Tamoxifen. A reduced number of invasive diagnostic procedures would decrease the risk of complications in patients, reduce the cost of medical care and may help to improve the detection of pathological hyperplasia of the endometrium. Further studies into the application and usefulness of elastography in investigating the endometrium in Tamoxifen-treated women, and possibly in other groups of patients, are thus reasonably warranted.

## 5. Conclusions

Previous studies conducted in the field of elastographic evaluations of the endometrium have focused on searching for absolute values that could numerically characterize the properties of this specific tissue [14,15,21,22]. In this study, the main objective was not the determination of the tissue properties, which, in elastography, may be affected by the examination itself, but rather, elastographic estimation of its quantity as expressed in the width of the softest endometrium layer. In women receiving Tamoxifen, 2D ultrasound screening does not give the expected results in determining the width of the uterine mucosa. In these patients, the studied elastographic examination scheme made it possible to minimize the number of diagnostic surgical procedures, avoid complications related to these procedures and reduce the cost of health care while maintaining the effectiveness of the diagnostic procedure. Thus, the use of elastography to measure the width of the softest endometrium layer in a population of women taking Tamoxifen appears to be a promising option for endometrial cancer screening.

## Figures and Tables

**Table 1:** Characteristics of studied patients.

Feature	Minimal Value	Maximal Value	Median
Age (years)	39	70	53
BMI	19.5	44	25.6
Duration of tamoxifen therapy [months]	6	108	22
Age at menarche	9	19	14
Parity	0	4	2

**Table 2:** The intraclass correlation coefficient (ICC; individual) for the two-way mixed-effects model, 95% confidence interval for this coefficient were calculated to analyze the consistency of agreement for the measurements carried out by the first and second physicians.

Investigator No. 1 vs. No. 2 Measurements	ICC	95% Confidence Interval
Ultrasound endometrial thickness	0.97	0.90; 0.99
Thickness of the softest endometrial layer in elastography	0.78	0.38; 0.94
Elastography index for the softest endometrial layer	0.75	0.31; 0.93
Elastography index for the endometrium-myometrium interface	0.43	−0.19; 0.81
Elastography index for the endometrium contour	0.72	0.26; 0.92
Length of the uterine cavity	0.92	0.72; 0.98
AP dimension of the uterine cavity	0.97	0.88; 0.99
Lateral dimension of the uterine cavity	0.77	0.36; 0.93
